# Supplementary material for: Comparison of Immunogenicity and Safety of Inactivated, Adenovirus-Vectored, and Heterologous Adenovirus-Vectored/mRNA Vaccines in Patients with Systemic Lupus Erythematosus and Rheumatoid Arthritis: A Prospective Cohort Study
Source: Vaccines (Basel). 2022 May 26;10(6):853. doi: 10.3390/vaccines10060853 (PMC9227480; doi:10.3390/vaccines10060853)
Supplement: Supplementary file 1 [file vaccines-10-00853-s001.zip › vaccines-1720457-supplementary.pdf]

Table S1. Number of participants according to the number of immunosuppressive drug used.

Number of immunosuppressive drug	AZD1222 (n = 43)	AZD1222/BNT162b2 (n = 28)	Inactivated (n = 23)
Zero (%)	0 (0%)	1 (3.6%)	0 (0%)
One (%)	7 (16.3%)	4 (14.3%)	0 (0%)
Two or more (%)	36 (83.7%)	23 (82.1%)	23 (100%)

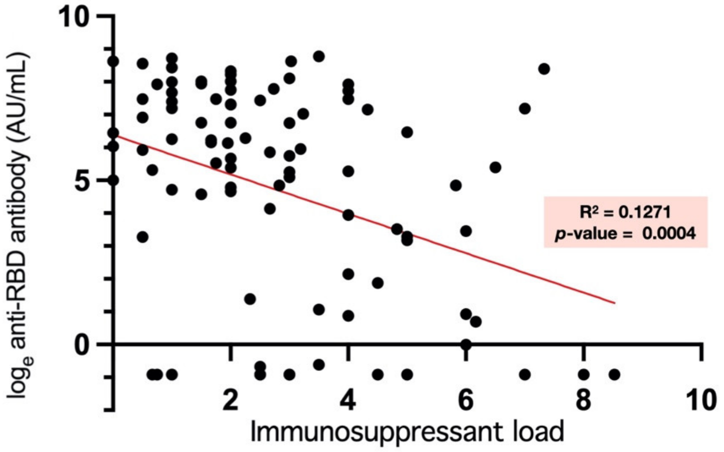

Figure S1. Relationship between total anti-RBD Ig level and ELISpot after the second vaccination.

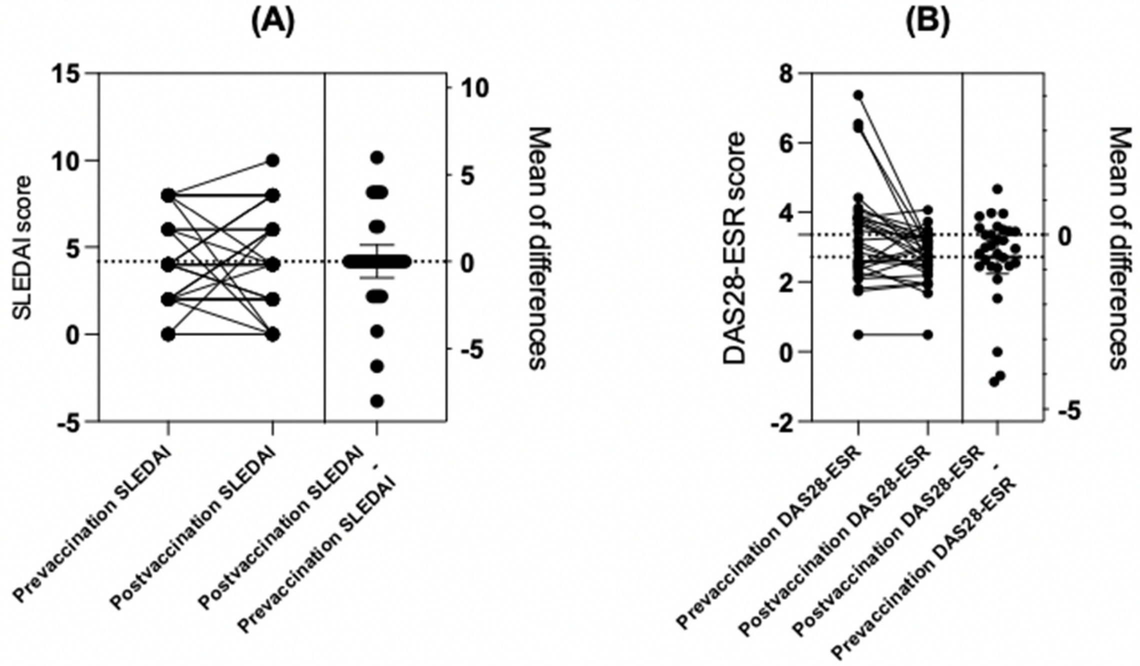

Figure S2. Estimation plot of the difference in disease activity scores between pre- and post-vaccination, grouped by (A) Systemic lupus erythematosus and (B) Rheumatoid arthritis.
